# Anxiety behavior and hypothalamic-pituitary-adrenal axis altered in a female rat model of vertical sleeve gastrectomy

**DOI:** 10.1371/journal.pone.0200026

**Published:** 2018-07-06

**Authors:** Alexandra R. Himel, Sharon A. Cabral, James P. Shaffery, Bernadette E. Grayson

**Affiliations:** 1 Department of Neurobiology and Anatomical Sciences, University of Mississippi Medical Center, Jackson, MS, United Status of America; 2 Department of Psychiatry and Human Behavior, University of Mississippi Medical Center, Jackson, MS, United Status of America; Technion Israel Institute of Technology, ISRAEL

## Abstract

Surgical weight loss results in a host of metabolic changes that culminate in net positive health benefit to the patients. However, the psychological impact of these surgeries has not been fully studied. On one hand, surgical weight loss has been reported to improve standard quality of life and resolution of symptoms of depression. But on the other hand, reports of self-harm and increased ER visits for self-harm suggest other psychological difficulties. Inability to handle anxiety following surgical weight loss has alarming potential ramifications for these gastric surgery patients. In the present study, we used models of diet-induced obesity and vertical sleeve gastrectomy (VSG) to ask whether anxiety behavior and hypothalamic-pituitary-adrenal (HPA) axis gene changes were affected by surgical weight loss under two diet regimens: i.e. low-fat diet (LFD) and high-fat diet (HFD). We show reduced exploratory behavior in the open field test but increased time in the open arms of the elevated plus maze. Furthermore, we show increased plasma levels of corticosterone in female VSG recipients in the estrus phase and increased levels of hypothalamic arginine-vasopressin (*avp*), pro-opiomelanocortin (*pomc*), and tyrosine hydroxylase (*th*). We report reduced dopamine receptor D1 (*drd1*) gene in prefrontal cortex (PFC) in VSG animals in comparison to Sham. Further we report diet-driven changes in stress-relevant gene targets in the hypothalamus (*oxt*, *pomc*, *crhr1*) and adrenal (*nr3c1*, *nr3c2*, *mc2r*). Taken together, these data suggest a significant impact of both surgical weight loss and diet on the HPA axis and further impact on behavior. Additional assessment is necessary to determine whether molecular and hormonal changes of surgical weight loss are the source of these findings.

## Introduction

Bariatric surgery for the long-term resolution of metabolic dysfunction is highly successful at achieving body weight loss, fat mass reduction, lipid improvements, and reduced need for diabetes medication [1–4]. The potential for these health improvements continues to result in greater numbers of surgeries with each passing year [5, 6]; many of the known negative side effects, inconveniences, and mortality risks of these surgeries have not altered the numbers of individuals receiving these surgeries [5]. There continue to be many unknowns concerning the long term behavioral implications of surgical weight loss. Though obesity itself has well-described effects on mental health status, a poorly understood area with respect to bariatric surgery is its effect on the mental well-being of the surgery recipients.

During the past decade, accumulating evidence suggests obesity has a significant association with various psychological disorders [7–16]. Links also exist between obesity/metabolic disorders and depression/anxiety disorders in adults [10, 17, 18]. In particular, adolescents and young adults suffering from severe obesity have greater levels of anxiety and depression [19–21] and risk of suicide [19]. Though a lack of mechanistic data exists in the cause of these disease processes, the relationship between psychological disorders issues and obesity are well documented.

If the rise in obesity during the last three decades is associated with the increased prevalence of psychological dysfunction, then one would predict that significant, durable weight loss by bariatric surgery should alleviate the adverse mental status potentially caused by obesity in these individuals. To date however, the data collected regarding the effect of bariatric surgery on psychological health has not clearly supported this hypothesis. Several concerning reports suggest that the incidence of self-harm emergency visits increases significantly after bariatric surgery as compared to prior to surgery [22–24]. The rates of self-harm post-surgery increased upwards of 5.7 fold in males and 7.4 fold in females in a Pennsylvania study [25]. In more recent studies emanating from Sweden, evidence of increased suicide and self-harm following surgical weight loss are reported in two separate Swedish cohorts although absolute numbers of harm incidents are minimal [26]. The positive improvements to anxiety reported in the early years after surgery appear to erode nearly a decade after surgical weight loss [27, 28]. Increased neuroticism, a feeling of lack of control and fear of intimacy are reported post-surgery [27, 28]. Since self-harm is linked with a history of anxiety [29], it is important to determine whether anxiety might play a role in the increased rate of self-harm after bariatric surgery. The gap in knowledge of how bariatric surgery affects mental health and behavior is an area of crucial importance to understand.

Previously, we reported that surgical weight loss in male rats alters the hypothalamic-pituitary-adrenal (HPA) axis in that *crh* is elevated in calorie-restricted and Roux-en-Y gastric bypass (RYGB) relative to Lean, Obese, and VSG [30]. We reported chronic stress-related behavior changes in that ACTH secretion was suppressed during a restraint test in VSG and RYGB male rats [30]. However, in these previous studies, we were not able to explore female HPA parameters and behavior and only tested males [30]. Since women seek bariatric surgery at an 8:1 ratio over males, in the current work we pursued psychological indices in female rats. In the present study, we used a female model of vertical sleeve gastrectomy and hypothesized that similar to males, surgically-induced weight loss would result in behavior and gene expression changes that suggest anxiety behavior and negative modulation of the HPA axis. Using open field testing (OFT) and the elevated plus maze (EPM), we measured exploratory and anxiety-related behavior and report gene changes in tissues of the HPA axis.

## Materials and methods

### Ethics statement

All procedures for animal use complied with the *Guidelines for the Care and Use of Laboratory Animals* by the National Institutes of Health. The University of Mississippi Medical Center Institutional Animal Care and Use Committee (IACUC) reviewed approved and supported this work in protocol 1423.

### Animals

Female Long Evans rats (200-225g) (Harlan, Indianapolis, IN) were initially multiply housed and maintained in a room on a 12/12-h light/dark cycle at 25°C and 50–60% humidity with *ad libitum* access to water. (Female rats were used because in humans females obtain surgery for weightless 8:1 in comparison to males.) After 1 week of acclimatization to the vivarium, female rats were placed on palatable high-fat diet (HFD) (#D03082706, Research Diets, New Brunswick, NJ, 4.54 kCal/g; 40% fat, 46% carbohydrate, 15% protein) for 4 weeks prior to surgery. Following 4 weeks on HFD average body weight was 282 ± 3.90 g. Animals were assigned to Sham-VSG or VSG groups in a counterbalanced fashion by body weight four days prior to surgery. Following the surgeries there were then 4 groups: 1) Sham-VSG continued on HFD (Sham-HFD) and were considered obese controls 2) Sham-VSG placed on LFD (Sham-LFD) (#D03082705, Research Diets, New Brunswick, NJ, 3.81 kcal/g, 9% fat, 76% carbohydrate, and 15% protein), 3) VSG continued on HFD (VSG-HFD) 4) VSG placed on LFD (VSG-LFD). In total, we used 25 rats for this study.

### Surgical procedures

#### Pre-operative care

Four days prior to surgery, body composition was assessed using an EchoMRI analyzer (Houston, TX). Animals were fed Osmolite OneCal liquid diet (Abbott Laboratories, IL) but no solid-food for 24 h prior to surgery.

*VSG/Sham-VSG*: We followed procedures as previously described [31]. VSG consisted of a midline abdominal laparotomy with exteriorization of the stomach. Ligaments and connective tissue were removed leaving an easily-articulated stomach. The lateral 80% of the stomach was excised using an ENDO GIA Ultra Universal stapler (#EGIAUSHORT, Covidien, MA) coupled with an ENDO GIA Auto Suture Universal Articulating Loading Unit, 45 mm–2.5 mm (#030454, Covidien, MA). A gastric tube in continuity with the esophagus and duodenum thus remained. This gastric sleeve was then reintegrated into the abdominal cavity and the abdominal wall was closed in layers using 4–0 coated vicryl suture (VE494, Ethicon, OH). The Sham-VSG mimics the VSG surgery including externalization of the stomach. However, in the case of the Sham, the stomach is gently pressed with forceps in the approximate area of the surgical resection and then immediately reintegrated into the abdomen which is then sutured and stapled.

#### Post-operative care

Following surgery, all rats received care for 3 d, consisting of once-daily subcutaneous injections of 3 mL sterile saline and 0.10 mL of carprofen (5mg/mL) and twice daily 0.10 mL Buprenex® (0.05mg/kg). Animals were maintained on Osmolite One Cal (Abbott Nutrition) until food was returned 3 d following surgery.

### Body weight, composition and food intake

Body weights were measured daily during the first post-operative week and then weekly throughout the remainder of the study. Echo Magnetic resonance imaging whole-body composition analysis (EchoMedical Systems, Houston, TX) was performed on all rats at 5 weeks post-surgery to determine fat and lean body composition.

### Behavior testing

#### Open Field Test (OFT)

On post-operative days 37–40 (POD37-40) animals were daily swabbed using a cotton tip applicator and cells from the lavage were rolled on glass slides. Using a microscope, lavage cytology was read to determine if the females were in estrus. For rats that were identified by cytological inspection to be in the estrus phase of the cycle, the rats were transported to the testing room 5 h into the light cycle and allowed to acclimate for an additional 1 h. The open-field unit (Columbus Instruments, Columbus, OH) consisted of 40 cm × 40 cm Plexiglass chambers embedded in individual cubicles (60 cm x 50 cm x 75 cm) open at the front [32–37]. The cubicles are equipped with a 6 watt overhead fluorescent light and testing was performed in a dimmed chamber of 6–8 lumens. 6 animals were tested simultaneously and placed in the arena for 20 min. Animal movements were tracked by the Columbus Automex software which uses an infrared beam grid detection system. The system was used to measure the following parameters: distance traveled over the maze and over the “center” area, time spent in the center area, total movement time (time in which the rat actively explored the maze, as any period where there is any movement of the rat), and number of rearings (vertical standing of rats on two hind legs). From these measured parameters, the distance ratio (distance traveled in central area/total distance traveled in the maze) and the rearings ratio (rearings made in central area/total number of rearings) were calculated.

#### Elevated plus maze (EPM)

On POD57-60 animals were daily swabbed by lavage and slides read to determine if the females were in estrus. If estrus was identified, animals were transported to the testing room, the rats were transported to the testing room 5 h into the light cycle and allowed to acclimate for 1 h. The EPM apparatus consisted of 4 arms (10 cm X 45 cm) of which 2 arms were enclosed in darkened walls 50 cm high and 2 arms had no enclosure at 90° angles to each other with all arm platforms elevated 50 cm from the floor. At the start of a trial, the rat was placed in the center with its nose directed toward the same open arm and allowed to explore the maze freely for 5 min. Testing was done using overhead fluorescent lighting allowing for a light range between 450–550 lux. Between each trial the EPM apparatus was cleaned with 70% ethanol and any feces removed. The total time spent, total distance covered, and distance in each arm and the center were digitally recorded by the Noldus Ethovision video tracking system (Noldus Ethovision, The Netherlands). Additional parameters determined in data analysis were latency to the open arm, average speed, as well as percentage of test time and distance spent in the open and closed arms

### Assays and gene expression

#### Plasma analytes

Plasma was diluted 1:10 in saline in order to measure corticosterone (#LS-F13013, LSBioscience) and triglycerides (#TR22421, ThermoFisher, VA). Blood glucose was measured using a hand-held glucometer (On Call Glucose Monitoring).

Euthanasia of animals. Animals were 6 h fasted the light phase and transported to a holding room 2 h prior to sacrifice by conscious decapitation with a guillotine. Tissues were harvested, weighed and flash frozen on methyl butane cooled dry ice and stored in -80°C until further processing

#### RNA processing and real-time PCR

For the medial basal hypothalamus (MBH) we dissected a hypothalamic cube bounded rostrally by the optic chiasm, caudally by the mammillary bodies, and dorsally at the apex of the third ventricle. Further we blocked the PFC using a hemisphere trimming the olfactory bulb and to approximately 2.52 mm with respect to bregma. RNA was extracted using a QIAGEN miniprep RNA kit (QIAGEN, Inc, Valencia, CA), and complementary DNA was transcribed using an iScript complementary DNA synthesis kit (Bio-Rad Laboratories, Hercules, CA). Quantitative polymerase chain reaction was performed on a Step-One Plus Real-Time PCR machine coupled with StepOne Software (v2.3) (Applied Biosystems) using TaqMan inventoried gene expression assays (Life Technologies, Foster City, CA) as listed in Table 2.

### Statistical analyses

All statistical analyses were performed using GraphPad Prism version 7.0 (GraphPad Software, San Diego, California) USA. To make time-wise comparisons, two-way ANOVA (variables: surgical group and time) were made. To make comparisons based on surgery and diet, two-way ANOVA with post hoc simple pairwise comparisons was used and F statistics reported. All results are given as means ± SEM. Results were considered statistically significant when *p* < 0.05.

## Results

### Body weight, food intake and body composition

**Fig 1:**
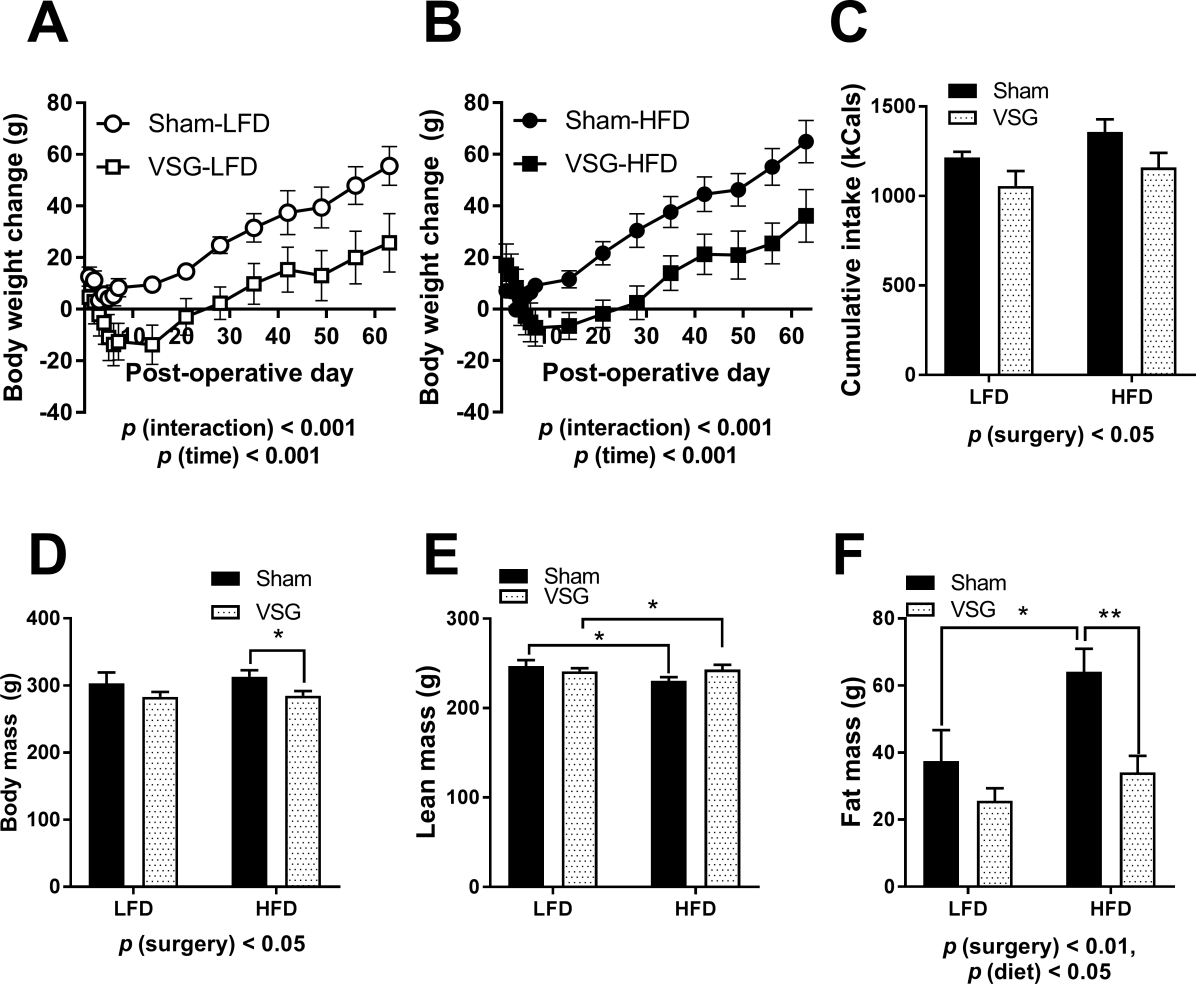
Metabolic parameters of female rats on LFD and HFD. Body weight following surgery for animals on **(A)** LFD and **(B)** HFD **(C)** Average daily food intake in kCal during the first 3 weeks after surgery. Body composition was measured at day 35 post surgery **(D)** Body mass **(E)** Lean mass **(F)** Fat mass. Data are presented as mean ±SEM. N = 5-7/group. *p <0.05, **p< 0.01.

Female rats showed body weight loss during the initial 7 post-operative days (POD) irrespective of the surgery. VSG animals lost a considerable amount of body weight over whether they were maintained on LFD (main effect of surgery, F (1, 160) = 46.89, *p* < 0.0001) (**Fig 1A**) or HFD (main effect of surgery, F (1, 160) = 38.37, *p* < 0.0001) (**Fig 1B**) following the surgery. Pairwise comparison show statistical significance at specific time points (**Fig 1A and 1B**). The VSG-LFD and VSG-HFD largely maintained their body weight loss during the course of the study but continued to grow in size after POD 14 (**Fig 1A and 1B**).

Average daily food intake was diminished during the first 14 POD resulting in a cumulative reduction in food intake over the course of the first 3 weeks (main effect of surgery, F (1, 19) = 5.801, *p* < 0.05) (**Fig 1C**) but food intake was normalized to the average intake of the Sham-LFD and Sham-HFD by POD21 (data not shown). At POD35, body composition was evaluated using EchoMRI. Body weight of the VSG-LFD and VSG-HFD at POD 35 was significantly reduced in comparison to Sham-LFD and Sham-HFD (main effect of surgery, F (1, 19) = 5.776, *p* < 0.05) (**Fig 1D**). Lean mass composition analysis showed no impact of surgery or diet in any of the groups (**Fig 1E**). VSG animals had reduced adiposity in comparison to Sham (main effect of surgery, F (1, 21) = 10.65, p < 0.01) (**Fig 1F**). However, HFD-fed animals, irrespective of surgery, had increased body fat in comparison to LFD-fed animals (main effect of diet, F (1, 21) = 7.457, *p* < 0.05) (**Fig 1F**).

### Open field test

**Fig 2:**
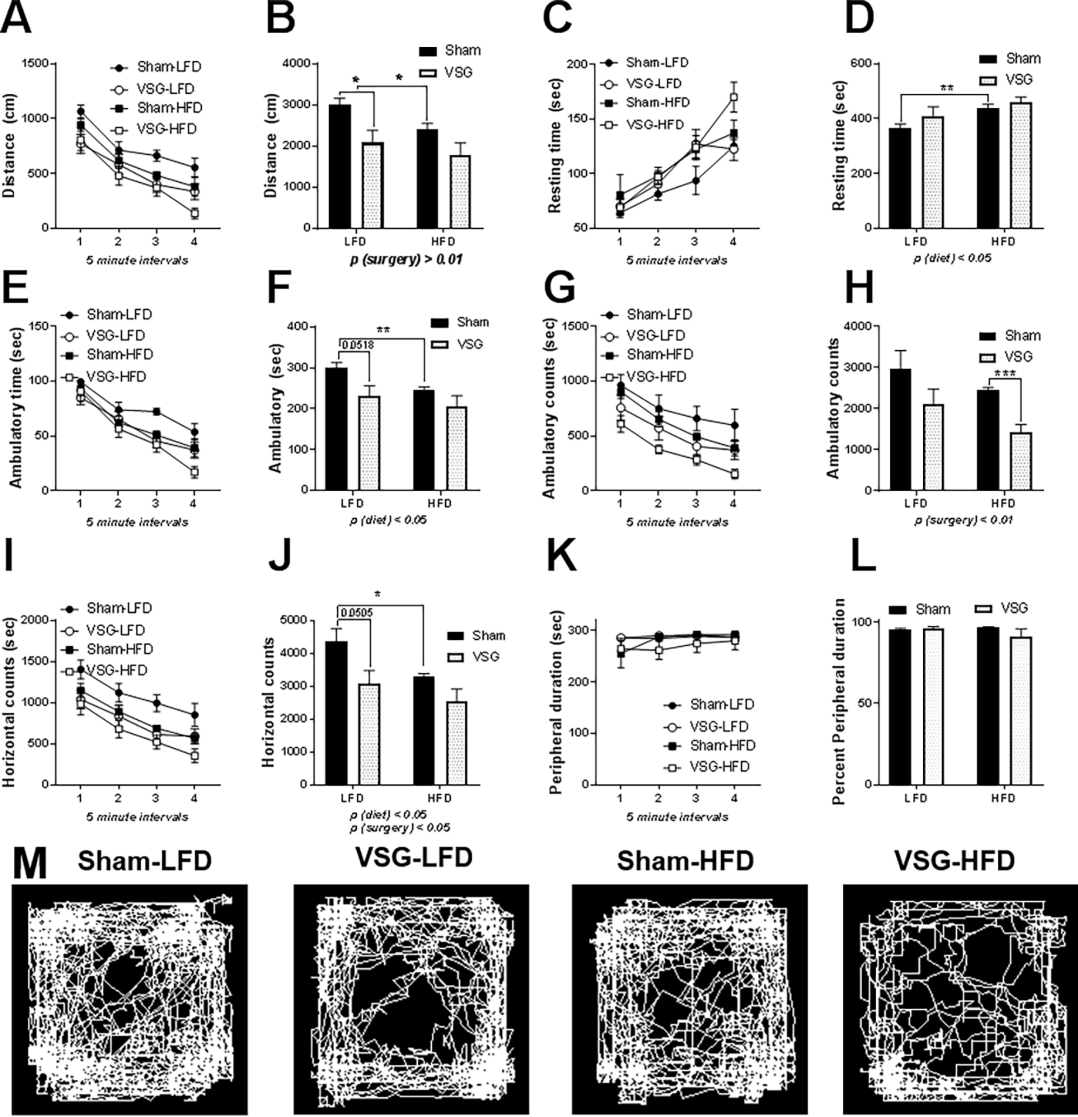
Behavioral analysis of female rats in open field test (OFT). **(A)** Distance travelled during each of the four 5 minute intervals **(B)** Cumulative distance travelled during the OFD (cm) during 20 min **(C)** Distance travelled during each of the four 5 minute intervals **(D)** Cumulative time spent resting in seconds over the 20 min testing period **(E)** Time spent ambulating during each of the 5 min intervals **(F)** Cumulative time ambulating in seconds over the 20 min test **(G)** Ambulatory counts reported for the 5 min intervals **(H)** Cumulative ambulatory counts for the 20 min test **(I)** Horizontal counts reported in 5 minute intervals **(J)** Cumulative horizontal counts for the 20 min test **(K)** Duration in sec spent in the periphery reported in 5 min intervals **(G)** Cumulative duration spent in the periphery (s) for the 20 min test. (M) Representative images of the activity of the animals during the 20 min interval. Cumulative data reported as 2ANOVA by surgery and diet. Data are presented as mean ±SEM. N = 5-7/group. *p < 0.05, **p < 0.01. ***p < 0.001.

On POD37-40, rats were lavaged daily to determine estrus phase of the cycle at beginning at 3 h after lights on. Animals who were positive for estrus cytology following inspection were moved into the testing room until performance in the Open Field Test (OFT) at 6 h after light on. Data are presented first in 5 minute intervals so that each interval can be inspected (**Fig 2 odd**) and assessed cumulatively for the 20 minute testing period for each of the measures (**Fig 2 even**. VSG rats travelled significantly less distance during the cumulative 20 min testing period (main effect of surgery, F (1, 20) = 9.639, *p* < 0.01) (**Fig 2A and 2B**). HFD-fed animals spent significantly increased time resting in comparison to LFD-fed animals (main effect of diet, F (1, 20) = 6.305, p < 0.05) (**Fig 2C and 2D**). However, VSG animals spent reduced time ambulating (main effect of surgery, F (1, 20) = 6.947, *p* < 0.05) (**Fig 1D**) (**Fig 2E and 2F**) and had significantly lower ambulatory counts (main effect of surgery, F (1, 20) = 9.341, *p* < 0.01) (**Fig 2G and 2H**) than the Sham animals. Additionally, the VSG animals exhibited reduced horizontal counts (main effect of surgery, F (1, 20) = 8.55, *p* < 0.01) (**Fig 2E**) than the Shams. Furthermore, there was an effect of diet (main effect of diet, F (1, 20) = 5.283 = 7.457, *p* < 0.05). (**Fig 2I and 2J**) where HFD-fed animal had a reduced number of horizontal counts compared to the LFD-fed animals. No significant differences were reported for the duration of time spent in the periphery (**Fig 2K and 2L**) of the OFT. There were no differences to report in stereotypic behavior, burst of stereotypic movement, vertical sensor counts, vertical sensor breaks, center duration, peripheral duration, % center entries, and % peripheral entries (data not shown).

### Elevated plus maze

**Fig 3:**
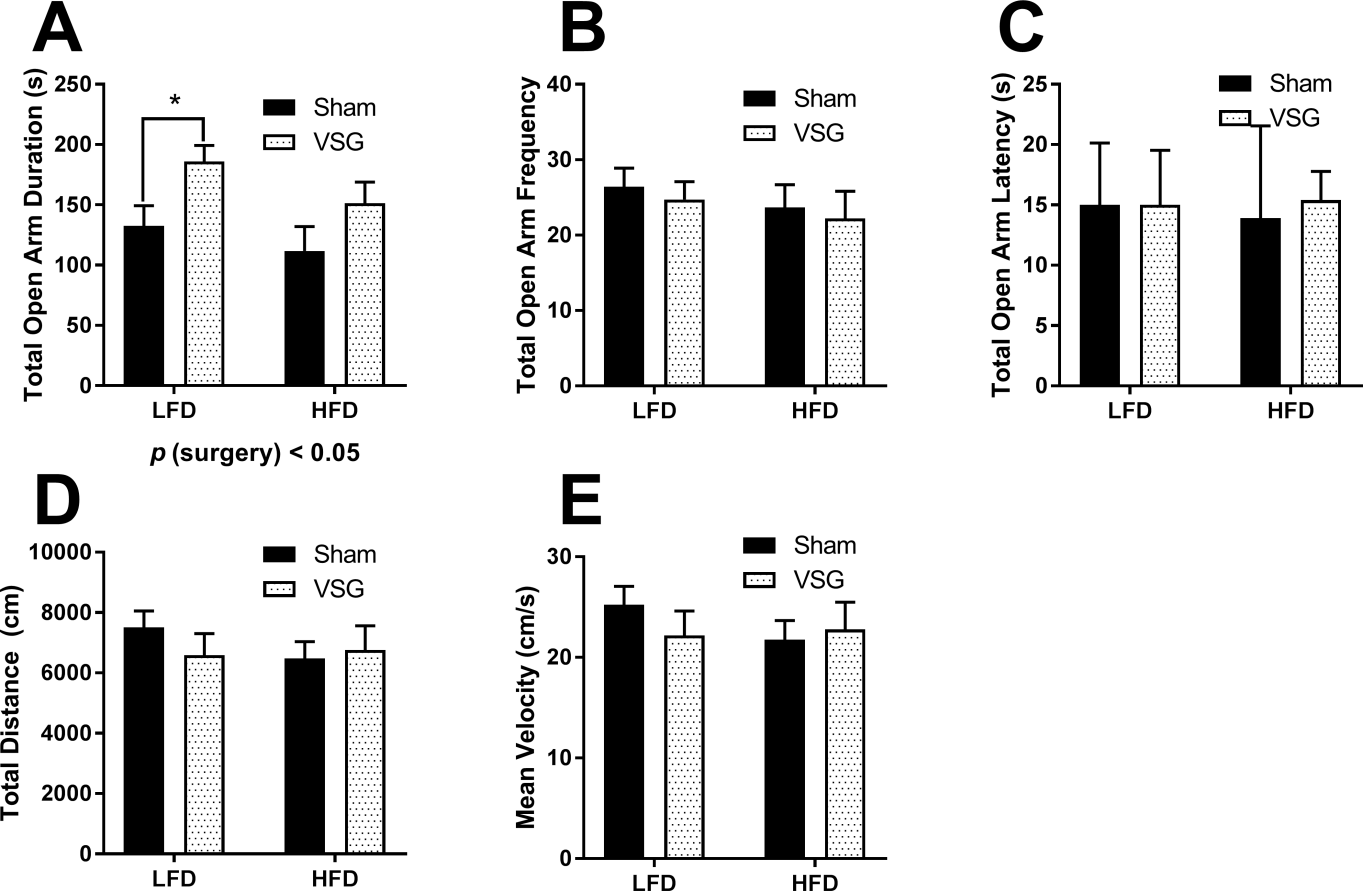
Behavioral analysis of female rats in elevated plus maze. **(A)** Total duration (s) of time spent in the open arms **(B)** Frequency of entries into the open arms **(C)** Latency to entry of the open arm **(D)** Total distance travelled during the EPM (cm) **(E)** Mean velocity in cm/s. Data are presented as mean ±SEM. N = 5-7/group. *p < 0.05, **p < 0.01. ***p < 0.001.

On POD57-60, rats were again lavaged daily to determine estrus phase of the cycle at 3 h after lights on. Animals who were positive for estrus cytology were moved into the testing room until performance in the elevated plus maze (EPM) at 5 h after light on. The VSG animals had a significantly longer duration of time spent in the open arm (main effect of surgery, F (1, 19) = 7.315, *p* < 0.05) (**Fig 3A).** There was no difference in the frequency of entries to the open arms (**Fig 3B)**; further there was no difference in the latency to enter the open arm (**Fig 3C).** In this test, there were no differences in the total distance travelled (**Fig 3D**) during the 5 min in the EPM nor the velocity of the travel (**Fig 3E**).

### Blood analytes

**Fig 4:**
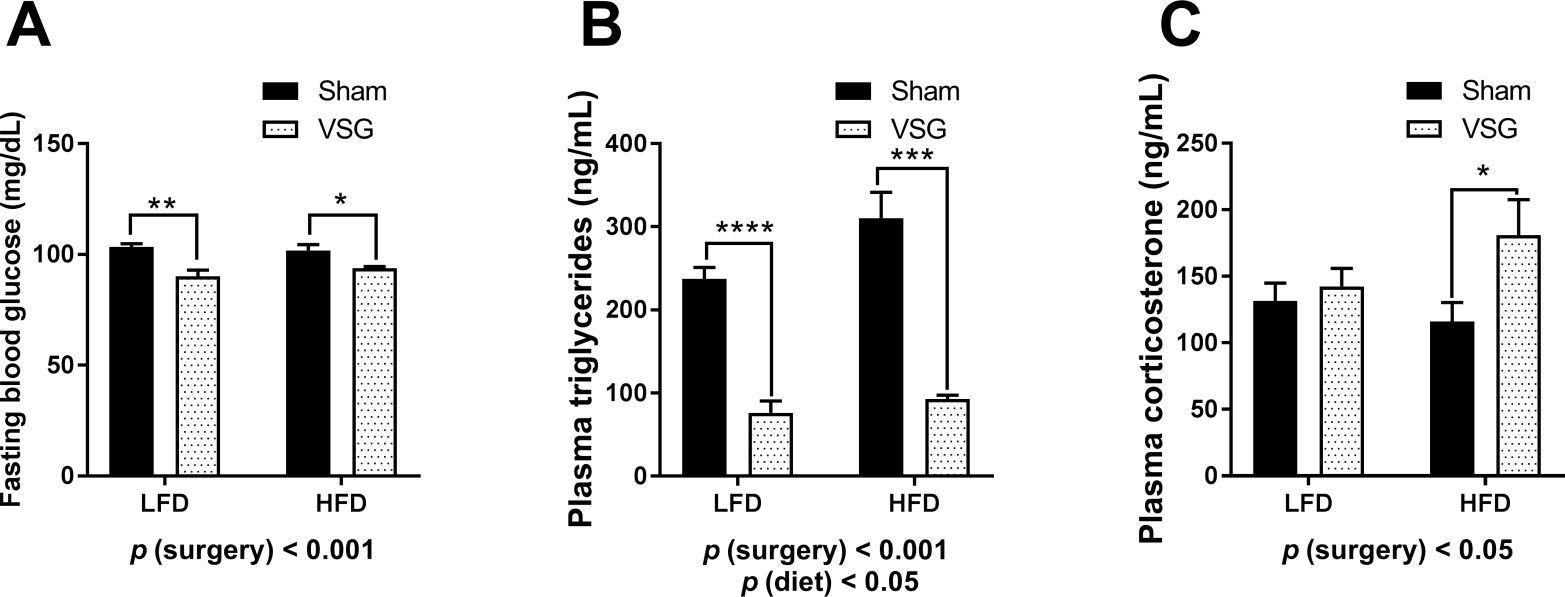
Plasma analytes in female rats. **(A)** Fasting plasma glucose **(B)** Fasting plasma triglycerides **(C)** Early light cycle corticosterone levels **(D)**. Data are presented as mean ±SEM. N = 5-7/group.

On POD66 blood glucose measured were made by tail vein sampling in 6 h fasted rats. Blood glucose was significantly reduced in VSG animals in comparison to Sham (main effect of surgery, F (1, 19) = 20.01, *p* < 0.001) (**Fig 4A**). Using these very same samples we measured a significant reduction in fasting triglycerides in VSG in comparison to Sham (main effect of surgery, F (1, 18) = 81.97, *p* < 0.001) and further an increase overall in HFD fed animals (main effect of diet, F (1, 18) = 4.548, *p* < 0.05) (**Fig 4B**). On POD74-75 tail vein blood was collected 2 h before lights off and corticosterone measured. VSG animals also exhibited higher corticosterone levels than Shams (main effect of surgery, F (1, 19) = 4.899, *p* < 0.05) (**Fig 4C**).

### Terminal tissue weights

On POD85/86, rats were euthanized by conscious decapitation and tissues harvested, weighed and analyzed. There was no significant difference in the mass of ovaries, uterus or thymus between groups (**Table 1**). VSG rats had a significant increase in heart mass in comparison to Sham animals (*p* (surgery) < 0.05) (**Table 1**).

**Table 1:** Terminal body weight raw organ weights.

		Sham-LFD			VSG-LFD			Sham-HFD			VSG-HFD			Statistics
		Mean		SEM	Mean		SEM	Mean		SEM	Mean		SEM	
ovary weight	(g)	0.066	±	0.007	0.063	±	0.004	0.066	±	0.003	0.059	±	0.003	* *
adrenal weight	(g)	0.022	±	0.005	0.025	±	0.002	0.023	±	0.002	0.022	±	0.003	* *
heart weight	(g)	1.076	±	0.056	1.249	±	0.084	1.131	±	0.024	1.265	±	0.102	*2ANOVA p (surgery < 0*.*05)*
uterine weight	(g)	0.681	±	0.124	0.602	±	0.038	0.610	±	0.046	0.839	±	0.108	* *
thymus weight	(g)	0.299	±	0.025	0.282	±	0.020	0.309	±	0.027	0.300	±	0.032	* *

Data are presented as mean ±SEM. N = 5-7/group.

### Gene expression for MBH, PFC, pituitary and adrenal

We processed the medial basal hypothalamus (MBH), prefrontal cortex (PFC), pituitary gland and adrenal gland for rtPCR detection to further explore changes. Within the MBH, we measured increased arginine vasopressin (*avp*) in VSG animals in comparison to Sham (main effect of surgery, F (1, 16) = 8.303, *p* < 0.05) (**Table 2**). We detected no change in corticotropin releasing hormone (*crh*) or receptor 1 (*crhr1*) but measured increased expression *crhr2* in animals fed a HFD (main effect of diet, F (1, 16) = 6.371, *p* < 0.05) (**Table 2**); there was no effect of surgery in the expression values. Hypothalamic oxytocin (*oxt*) gene expression was increased in HFD-fed animals in comparison to LFD-fed animals (main effect of diet, F (1, 16) = 10.03, *p* < 0.01) (**Table 2**). We further found trends towards increased pro-opiomelanocortin (*pomc*) expression due to VSG surgery (main effect of surgery, F (1, 18) = 4.156, p = 0.0565) (**Table 2**). Finally in the MBH we measured trends towards increased tyrosine hydroxylase (*th*) expression as a function of VSG surgery suggestive of increased hypothalamic dopamine production (main effect of surgery, F (1, 16) = 4.175, *p* = 0.0578) (**Table 2**).

**Table 2:** Gene expression analysis of the medial basal hypothalamus, prefrontal cortex, pituitary and adrenal gland. Data are presented as mean ±SEM. N = 5-7/group.

		Sham-LFD			VSG-LFD			Sham-HFD			VSG-HFD			Statistics
***Medial basal hypothalamus***														
***avp***	Rn00690189_g1	100	±	21.94	178.2	±	37.59	147.4	±	34.78	271	±	19.43	*2ANOVA p (surgery) < 0*.*05*
***crh***	Rn01462137_m1	100	±	13.63	119.7	±	17.07	135.2	±	13.59	135.8	±	20.45	* *
***crhr1***	Rn00578611_m1	100	±	14.74	129.8	±	11.46	115.3	±	17.89	140.1	±	7.748	* *
***crhr2***	Rn00575617_m1	100	±	10.56	119.1	±	7.78	138.2	±	16.25	145.7	±	5.781	*2ANOVA p (diet) < 0*.*05*
***nr3c1 ***	Rn00561369_m1	100	±	35.32	107.8	±	19.4	87.41	±	24.8	50.61	±	23.21	* *
***oxt***	Rn00564446_g1	100	±	11.39	107.8	±	11.03	150.5	±	13.01	141.1	±	16.35	*2ANOVA p (diet) < 0*.*05*
***pomc***	Rn00595020_m1	100	±	21.94	178.2	±	37.59	147.4	±	34.78	271	±	19.43	*2ANOVA p (surgery) = 0*.*0565*
***th***	Rn00562500_m1	100	±	13.12	158.9	±	37.97	77.1	±	10.55	132.9	±	36.77	*2ANOVA p (surgery) = 0*.*0578*
***Pre-frontal cortex***														* *
***nr3c1 ***	Rn00561369_m1	100	±	23.53	71.89	±	10.23	51.42	±	8.89	50.6	±	3.67	*2ANOVA p (diet) < 0*.*05*
***drd1***	Rn03062203_s1	100	±	31.4	53.24	±	12.02	92.56	±	23.61	40.71	±	3.94	*2ANOVA p (surgery) = 0*.*0523*
***ht2cr***	Rn00562748_m1	100	±	21.2	88.55	±	13.17	83.86	±	16.11	84.97	±	10.05	* *
***Pituitary***														* *
***pomc***	Rn00595020_m1	100	±	18.19	114.7	±	19.47	138.6	±	22.26	81.37	±	13.85	* *
***crhr1***	Rn00578611_m1	100	±	16.23	120.3	±	9.219	158.0	±	16.23	94.42	±	11.74	*2ANOVA p(interaction) < 0*.*05*
***crhr2***	Rn00575617_m1	100	±	32.44	211.3	±	60.69	134.0	±	30.02	130.7	±	38.42	* *
***nr3c1 ***	Rn00561369_m1	100	±	15.6	115.9	±	18.84	115.2	±	9.187	78.44	±	78.44	
***Adrenal***														* *
***nr3c1 ***	Rn00561369_m1	100	±	15.25	136.3	±	30.21	153.3	±	18.55	206.4	±	49.88	*2ANOVA p(diet) = 0*.*0518*
***nr3c2***	Rn00565562_m1	100	±	8.626	177.4	±	53.26	196.6	±	49.62	301.3	±	57.87	*2ANOVA p(diet) < 0*.*05*
***mc2r***	Rn01491505_m1	100	±	15.91	120.7	±	51.38	165.1	±	42.59	339.6	±	74.52	*2ANOVA p(diet) < 0*.*01*

Data are presented as mean ±SEM. N = 5-7/group.

We measured some expression changes in the prefrontal cortex (PFC). We report reduced glucocorticoid receptor (*nr3c1*) as a function of diet (main effect of diet, F (1, 17) = 5.492, *p* < 0.05). We further measured trends towards increased dopamine receptor 1 (*drd1*) expression as a function of surgery, (main effect of surgery, F (1, 16) = 4.393, *p* = 0.0523). We report no change in serotonin 2C receptors (*ht2cr*) in the PFC.

We further determined no changes among groups to *pomc* and *crhr2* in mRNA from the pituitary gland and an significant interaction in the expression of *crhr1* (interaction of surgery and diet, F (1, 18) = 11.99, *p* < 0.01) (**Table 2**) in which gene expression is increased in VSG-LFD in comparison to Sham-LFD, but reduced in VSG-HFD in comparison to Sham-HFD. Though there were no main effects of surgery or diet in *nr3c1* expression in the pituitary.(**Table 2**).

In the adrenal gland, we measured increases in glucocorticoid receptor (*nr3c1)* in the HFD-fed rats (main effect of diet, F (1, 18) = 4.337, *p* = 0.0518), mineralocorticoid receptor (*nr3c2)* (main effect of diet, F (1, 18) = 4.907, *p* < 0.05) and melanocortin 2 receptor (*mc2r*) (main effect of diet, F (1, 17) = 8.527, *p* < 0.01) in HFD-fed animals in comparison to LFD-fed animals (**Table 2**).

## Discussion

The overall goal of this study was to determine if female rats who have undergone the most common surgical weight loss procedure, VSG, show behavioral or gene expression changes suggestive of a dysregulated HPA axis. Previously we tested male rats having had either VSG, RYGB or having been calorically-restricted to a lower body weight comparing them to obese controls and found evidence that the HPA axis was negatively affected by the surgeries [30]. Our present study results show evidence in female rats having received VSG on two diets that there are some altered anxiety behaviors, increases in corticosterone and changes in HPA gene expression after surgical weight loss.

### VSG females have metabolic improvements whether on LFD or HFD

In the present study, VSG was effective in reducing body weight in female rats, as previously reported [30, 38, 39], whether maintained on a LFD or HFD post-surgery. VSG maintained a reduced body weight, body fat and body fat percentage as a result of the VSG. The fasting blood glucose and fasting plasma triglyceride levels of VSG rats were significantly reduced in comparison to Sham. Together, these data demonstrate that the cohort exhibits the characteristic signs of VSG-induced metabolic improvements that have been previously reported [39–41].

### VSG females have increased plasma corticosterone

In the present work, we report increased plasma corticosterone levels in female rats having received VSG in comparison to Sham suggestive of a potential change in the stress axis. In our previous work, when a restraint was performed in male rats, we observed elevated morning corticosterone in rats having received RYGB but no differences in corticosterone levels in VSG rats in comparison to Shams. We also observed a depressed ACTH release in both VSG and RYGB following the restraint suggestive of chronic stress in the male bariatric rats [30]. These collective data led to our interest to test anxiety behavior in VSG females particularly as VSG is the most commonly sought surgical means of weight loss [6].

### VSG females exhibit reduced exploratory behavior in the OFT

Here we report behavior changes in the Open Field Test (OFT). Without respect to surgery or diet, all the females preferred the periphery of the open field rather than the center, spending greater than 95% of the time in the periphery. Furthermore, between the surgical and diet groups there were no differences in the duration of time or percentage of time spent in either the periphery or the center. If the use of the OFT is limited to the distinction between time spent in the periphery and center, there were no differences identified in anxiety behavior between the various groups. One aspect of utility in tracking locomotor behavior in an open environment such as the OFT lies in the ability to discern if animals engage in reduced locomotion and associated exploratory behaviors [42]. Thus reduced locomotion in the OFT potentially suggests attempt to evade a predator through freezing and thus can be further used as a tool to infer anxiety [42]. The VSG females in estrus did spend overall less time ambulating in the open field and ambulated a shorter distance in the 20 min testing time. The VSG females also exhibited less horizontal beam break counts, i.e. collectively representing behaviors such as rearing and stretching, suggestive of overall reduced emotionality [43]. These indices suggest that exploratory behavior is reduced in the VSG females in comparison to the Sham females irrespective of diet.

### VSG females display anxiolytic behavior in the EPM

The behavior of the female rats in estrus in the Elevated Plus Maze (EPM) is not directly congruent with the behavior observed in the OFT. When tested several weeks later in the EPM, the VSG females in estrus exhibit preference for the open arms of the EPM, spending overall a greater amount of time in the open arms and a greater overall percentage of time in the open arms with no difference in the frequency of entries or the latency to enter the open arms. In comparison to the OFT, where animals explored less as evidenced by overall less time and distance ambulating, there was no difference observed in the distance or velocity of the rats within the EPM. However, the time was spent specifically in the open area of the maze and not the enclosed darker area. Normally, this behavior is interpreted as exhibiting reduced anxiety [44, 45].

The incongruency we observe between these two tests is not unprecedented in the assessment of anxiety-related behavior. [46]. Anxiety behavior is multi-dimensional and can be situation- and test- specific. Furthermore, input from limbic regions of the brain such as the amygdala, prefrontal cortex and hippocampus during performance of the tasks in tests like the OFT and EPM can alter the functional behavior elicited from each of the test [46]. As these were females, we did control for the day of the estrus cycle. However, the OFT and EPM spanned several weeks and conceivably remodeling of the brain and other changes continue to occur following surgical weight loss. This is somewhat anticipated based on reports of behavior in human outcomes of bariatric surgery. For instance, there are some reports of some reduced anxiety after bariatric surgery [47, 48]. Whereas RYGB and VSG in humans weight loss appears to improve quality of life [49, 50] and reduce levels of depression [51, 52], when anxiety is probed specifically, there are some short-term improvements [47, 53, 54] or no significant change [48, 52, 53, 55] or even a decline of the anxiolytic improvements of surgery over time [54]. Therefore the timing of the tests and tissue measurements with we performed in these studies with respect to the timing of the surgery could further complicate our current interpretations.

### VSG alters some HPA axis changes that display stress and anxiety

We performed quantitative analysis of mRNA extracted from HPA axis-relevant tissues, i.e. hypothalamus, pre-frontal cortex, hippocampus, pituitary and adrenal gland tissues, to determine if relevant gene changes might shed light on the increased corticosterone and behavior changes following VSG. The VSG females showed no changes in hypothalamic *crh* mRNA, but exhibited a significant increase in hypothalamic *avp* mRNA. This same pattern of no altered *crh* and increased *avp* was also reported in our study on the HPA axis in male rats in both VSG and RYGB [30]. Since in the MBH, *crh* and *avp* are co-expressed in the same neurons, increases in *avp* mRNA have been linked with increased stress reactivity [56] leading to increased release of ACTH and downstream elevations in release of corticosterone [56]. AVP also integrates blood pressure responses. With elevations in both VSG males [30] and females, one might hypothesize VSG animals have increased blood pressure. In fact, females having undergone VSG have either no change or reduced blood pressure both prior to pregnancy and during gestation [57].

In the present study, we did not observe altered pituitary *pomc* which would have indicated enhanced ACTH release but we did measure an increase in hypothalamic *pomc* mRNA in VSG animals in comparison to Sham. The *pomc* gene codes for the production of several circulating hormones including α melanocyte-stimulating hormone (αMSH) which acts as an anorexigen and may be involved in suppressing food consumption in these VSG females. This elevation in hypothalamic *pomc* was absent in our previous study in male rats having received VSG and may be a reflection of the amount of time the females were fasted prior to euthanasia [30]. Furtherthere was no effect by diet or surgery on pituitary *nr3c1* (GR) expression. Accordingly, we report significantly increased light cycle plasma corticosterone levels in VSG females, particularly in VSG-HFD females. We were not able to measure circulating ACTH in the present body of work, but previously in males, it was not altered following VSG surgery [30].

In the present work we report increased hypothalamic *th* gene expression in VSG animals. *Th* is the rate-limiting enzyme for dopamine production. Altered dopaminergic signaling has been shown between the gut and ventral striatum of male RYGB rats and thought to be responsible for altered fat intake after surgery [58]. This work has not been replicated however with VSG animals. Here we also report a down-regulation of *drd1* in the PFC of VSG female rats, potentially in response to increased dopamine signaling. This reduction in D1 dopamine receptors may contribute to the altered anxiety behavior in our female rats. The caveat to our PFC measurements is that we used the whole block rather than microdissecting sub-regions. It is possible that regional opposing differences cancelled out our ability to discern changes. Obesity results in altered dopamine signaling in the limbic brain structures that appears to modulate overall stress and anxiety behaviors [59–62].

Overall, gene expression changes in HPA-relevant tissues were far more impacted by diet than surgery. Elevations in oxytocin mRNA expression in HFD-fed females may be influences by increased circulating leptin levels that are elevated with high-fat feeding [63]. However, in bariatric surgery, there are reductions in overall leptin levels due to the substantial loss of adipose tissue [64]. Nonetheless, leptin reduced in VSG females due to loss of body fat mass [41].

Our previous published data in males suggested that rats that received bariatric surgery, in particular RYGB and to a lesser extent VSG, had reduced thymic weight and increased adrenal weight which we interpreted as a consequence of chronic stress [30]. Here we see no impact of either diet or surgery on thymic and adrenal weight in VSG females and so the concerns of chronic stress phenotypes are diminished. However, in the current study we observed increased total heart mass and when normalized to body weight. With respect to the heart weight, we did measured trends towards reductions in blood pressure in VSG female rats non-pregnant and pregnant rats [65]. The significance of the increased heart weight is not apparent.

Taken together, we have determined that female rats having undergone VSG do exhibit altered behavioral measures (reduced exploratory behavior, increased open arm time) and gene changes (*avp*, *pomc*, *th*) suggestive of an impact of surgery on the HPA axis. In addition, we report increased plasma corticosterone after VSG. In our study, we tested wild-type rats without genetic manipulation. The human data collectively suggests that pre-surgical psychological vulnerabilities of a variety of polygenic root causes may influence post-surgical behavior status [66]. Therefore to extend this work in rodent models, it would be useful to incorporate animal models which that recapitulate some of these genetic root causes might help with understanding the psychopathology of these diseases of mental health. Taken together, these underlying psychological issues may be altered and further exacerbated by psychological changes that occur as a result of surgical weight loss manipulations.
